# Screening for *THAP1* Mutations in Polish Patients with Dystonia Shows Known and Novel Substitutions

**DOI:** 10.1371/journal.pone.0129656

**Published:** 2015-06-18

**Authors:** Ewa Golanska, Agata Gajos, Monika Sieruta, Malgorzata Szybka, Monika Rudzinska, Stanislaw Ochudlo, Tomasz Kmiec, Pawel P. Liberski, Andrzej Bogucki

**Affiliations:** 1 Department of Molecular Pathology and Neuropathology, Medical University of Lodz, Lodz, Poland; 2 Department of Movement Disorders, Medical University of Lodz, Lodz, Poland; 3 Department of Neurology, Medical University of Silesia, Central Clinical Hospital, Katowice, Poland; 4 Stroke Department and Department of Neurology, Central Clinical Hospital, Katowice, Poland; 5 Child Neurology Department, The Children’s Memorial Health Institute, Warsaw, Poland; University of Pennsylvania Perelman School of Medicine, UNITED STATES

## Abstract

The aim of this study was to assess the presence of DYT6 mutations in Polish patients with isolated dystonia and to characterize their phenotype. We sequenced *THAP1* exons 1, 2 and 3 including exon-intron boundaries and 5’UTR fragment in 96 non-DYT1 dystonia patients. In four individuals single nucleotide variations were identified. The coding substitutions were: c. 238A>G (p.Ile80Val), found in two patients, and c.167A>G (p.Glu56Gly), found in one patient. The same variations were present also in the patients’ symptomatic as well as asymptomatic relatives. Mutation penetration in the analyzed families was 50-66.7%. In the fourth patient, a novel c.-249C>A substitution in the promoter region was identified. The patient, initially suspected of idiopathic isolated dystonia, finally presented with pantothenate kinase 2-associated neurodegeneration phenotype and was a carrier of two *PANK2* mutations. This is the first identified NBIA1 case carrying mutations in both *PANK2* and *THAP1* genes. In all symptomatic *THAP1* mutation carriers (four probands and their three affected relatives) the first signs of dystonia occurred before the age of 23. A primary localization typical for DYT6 dystonia was observed in six individuals. Five subjects developed the first signs of dystonia in the upper limb. In one patient the disease began from laryngeal involvement. An uncommon primary involvement of lower limb was noted in the *THAP1* and *PANK2* mutations carrier. Neither of these *THAP1* substitutions were found in 150 unrelated healthy controls. To the contrary, we identified a heterozygous C/T genotype of c.57C>T single nucleotide variation (p.Pro19Pro, rs146087734) in one healthy control, but in none of the patients. Therefore, a previously proposed association between this substitution and DYT6 dystonia seems unlikely. We found also no significant difference between cases and controls in genotypes distribution of the two-nucleotide -237-236 GA>TT (rs370983900 & rs1844977763) polymorphism.

## Introduction

*THAP1* encodes a transcription factor named THAP domain containing apoptosis-associated protein 1. Its DNA-binding properties are associated with the N-terminal THAP domain including a zinc-finger structure [1]. Over 90 point mutations within all three *THAP1* exons have been described in DYT6 dystonia patients of different ethnicities. A majority of the variations was found in the THAP domain [2]. *THAP1* mutations are inherited in an autosomal dominant manner with penetrance of 40–60% [3].

DYT6 dystonia typically presents as early-onset progressive disorder with prominent cranio-cervical and upper limb muscles involvement. Speech impairment being a result of laryngeal dystonia is quite common. Rostrocaudal spreading of dystonic movements is usually observed. Nonetheless, DYT6 phenotype is highly variable ranging from unaffected carriers to severe generalized dystonia, even within a single family [4–8].

In this study we investigated a cohort of Polish patients with idiopathic isolated dystonia to assess the prevalence of *THAP1* mutations and to characterize their phenotype. We describe four patients carrying *THAP1* variations, including one novel point substitution within the promoter region.

## Materials and Methods

### Subjects

The *THAP1* gene was analyzed in a group of 96 DYT1-negative patients with: (1) early-onset (<30 years) generalized dystonia; or (2) focal or segmental dystonia localized in upper limbs or neck (including isolated task specific dystonias), with or without positive family history of dystonia, independently of age of onset. The study population is characterized in Table 1.

**Table 1:** Clinical characteristics of the study population.

Patients		Total	Focal	Task specific	Segmental	Generalized/multifocal
No (sex: male/female)		96 (46/50)	31(16/15)	9 (2/7)	13 (6/7)	43(22/21)
Median age of onset: years (range)		19 (1–66)	26.5 (7–51)	32.5 (18–66)	23 (6–62)	10 (1–63)
Median duration of disease: years (range)		11 (1–50)	9 (1–34)	33 (3–22)	11.5 (3–44)	15.5 (1–50)
Median age at examination: years (range)		35 (6–78)	37 (19–78)	45 (32–70)	37 (23–72)	29 (6–67)
**Localization of dystonia at onset**	Cervical	30	18		7	5
	Upper extremities	47	12	9	6	20
	*writer’s cramp*			*8*		*1*
	*musician dystonia*			*1*		
	Lower extremities	13	1			12
	Trunk					
	Blepharospasm	1				1
	Oromandibular	1				1
	Pharyngeal	1				1
	N/A	3				3
**Localization of dystonia at examination**	Cervical	59	18		12	29
	Upper extremities	72	12	9	8	43
	Lower extremities	36				36
	Trunk	27			2	25
	Blepharospasm	7			2	5
	Oromandibular	16			5	11
	Pharyngeal	19			1	18
Family history n (%)		35 (36.4)	13 (72.1)	0 (0.0)	5 (38.5)	17 (39.5)

The data does not include family members of the probands.

All patients were recruited from Polish movement disorders centers. The patients were examined by movement disorders specialists. In all subjects neuroimaging studies were done. The exposure to neuroleptics or toxic substances was excluded based on the medical history. The laboratory tests and slit-lamp examination were performed to rule out Wilson’s disease as the cause of dystonia. In all patients the DYT1 mutation (GAG deletion in *TOR1A* exon 5) was excluded as described before [9].

As controls, we analyzed 150 neurologically healthy individuals (53.3% women), median age 46 years (range 20–86 years).

### Ethics statement

The protocol of the study was approved by the Bioethical Committee at the Medical University of Lodz (permit No: RNN/176/09/KE, 14.07.2009). Written informed consent has been obtained from all examined individuals.

### Methods

*THAP1* exons 1, 2 and 3 including exon-intron boundaries and 5’UTR fragment were PCR-amplified and sequenced as described elsewhere [10]. The presence of the identified mutations was additionally confirmed with PCR-RFLP method with the use of Hpy188I, New England BioLabs (Glu56Gly); SspI, Thermo Scientific (Ile80Val); and BccI, New England Biolabs (-249C>A) restriction enzymes.

The significance of the coding substitutions was predicted with PolyPhen-2 HumVar model (http://genetics.bwh.harvard.edu/pph2/) [11,12], SIFT (http://sift.jcvi.org/) [13] and Mutation Taster (http://www.mutationtaster.org/) [14] algorithms. Fisher’s exact test was used to estimate a potential difference between the patients and controls in the distribution of *THAP1* -237-236 GA>TT (rs370983900&rs1844977763) genetic polymorphism.

## Results

Four carriers of heterozygous sequence variants were identified. These were: c. 238A>G, p.Ile80Val substitution (found in two patients); c.167A>G, p.Glu56Gly change (in one patient); and one novel non-coding -249C>A substitution, located 5’ of the exon 1 (in one patient). Variants accounted for 4.2% (4/96) of all analyzed cases. None of these substitutions was found in healthy controls. Clinical data of the patients with *THAP1* mutations are presented in Table 2.

**Table 2:** Clinical characteristics of *THAP1* mutations carriers.

Case		Patient 1	Patient 2	Patient 3	Patient 4
Mutation		exon 2, c.238A>G, (p.Ile80Val)	exon 2, c.238A>G, (p.Ile80Val)	exon 2, c.167A>G, (p.Glu56Gly)	Promoter, c.-249C>A
Sex		M	M	M	M
Age at onset		7	23	10	10
Age at last examination		29	51	56	20
Family history		Positive	Positive	Positive	Negative
**Site at onset**	Upper limb	+	+		
	Lower limb				+
	Larynx			+	
**Site at examination**	Upper limb	+	+	+	+
	Lower limb	+	+	+	+
	Trunk			+	+
	Cervical	+	+	+	+
	Larynx			+	+
	Face			+	+
Family members with the same mutation		mother (A), sister (S)	daughter (A)*, son (S)*	paternal uncle (S), twin sister (A), son (A)	father (A)

(A), asymptomatic; (S), symptomatic;

*, genetic testing of the Patient’s 2 relatives was performed in Department of Neurology, University of Tennessee Health Science Center, Memphis, USA [15].

### Patient 1

A 29-year-old man with the Ile80Val substitution developed writer’s cramp as the first sign of dystonia at the age of 7. In the following years his right arm dystonia lost its task-specificity. When he was a teenager the dystonic movements spread to the left arm and to the lower limbs. At the age of 29, apart from dystonic movements involving his upper and lower limbs, the patient demonstrated mild laryngeal dystonia. The Ile80Val substitution was also found in his mother and sister. The proband’s mother is 51 years old and she remains asymptomatic. His 23-year-old sister developed mild dystonia and tremor of the right hand at age of 13, the progression of symptoms in the following years was rather slight and dystonia was limited to the right arm.

### Patient 2

A 51-year-old male carrier of the Ile80Val substitution noted the first signs of writer’s cramp on the right side at the age of 23. Then the right arm dystonia became non-task specific. At the age of 45 right rotational torticollis with dystonic tremor developed. A few months later, bilateral dystonic tremor of upper limbs was also observed. The patient reported also a tremor of the right leg caused by pressing the car pedal while driving.

The paternal grandmother of the patient developed head tremor when she was about 50 years old. It persisted as an isolated symptom until her death at the age of 80. Two of the proband’s three children were *THAP1* Ile80Val mutation positive. The daughter was a non-affected carrier. The youngest son developed asymmetric (R>L) tremor of limbs at 20 years of age, then dystonic movements occurred distally in his right upper limb. The proband was diagnosed in two Polish movement disorders centers; his genotyping was performed independently in Department of Molecular Pathology and Neuropathology, Medical University of Lodz, Poland and in the Department of Neurology, University of Tennessee Health Science Center, Memphis, USA. The patient and his family were a part of material presented by LeDoux et al. [15].

### Patient 3

A 56-year-old man developed a strained voice at the age of 10. While he was in his twenties, jerky movements appeared first in the right and then in the left arm. At the age of 40 he noted difficulties when writing with the right hand. Eight years later he developed writer’s cramp on the opposite side. At the age of 52, abnormal head posture and gait disturbances with feeling of tension in the right calf and tendency to stumble appeared. On examination, at the age of 53 years (and followed up for 3 years), the patient presented with mild blepharospasm, anterocollis with the pronounced platysma involvement, dystonic movements and postures involving both upper limbs, feet and trunk. Asymmetrical (R>L) jerky, large amplitude tremor of both arms was observed.

The same nucleotide variation was found in his three relatives: a symptomatic 70-year old paternal uncle and two asymptomatic carriers (56-year-old dizygotic twin sister and 29-year-old son). The uncle of the patient developed right-sided writer’s cramp at the age of 20 and 20 years later on the opposite side; no other dystonic movements were observed. The deceased great paternal grandfather of the proband (not tested for *THAP1* mutations) probably suffered from writer’s cramp. For more specific information about the patient and his family history see our previous report [10].

### Patient 4

A 20-year-old man developed the first signs of dystonia when he was 10 years old. Initially dystonic movements appeared in one foot and after several weeks the opposite foot was involved. For a few consecutive years dystonic movements were limited to the lower limbs, therefore a test for DYT1 mutation (GAG deletion in *TOR1A* exon 5) was done first. The screening for DYT6 nucleotide changes was done as a routine procedure after exclusion of DYT1 mutation. *THAP1* sequencing demonstrated a novel -249C>A substitution. A sudden disease progress took place and retrocollis and extensor truncal and neck dystonia (dystonic opistotonus) developed. At the age of 16 the neurological examination revealed generalized dystonia involving all extremities, trunk, neck and face, moreover the spastic paraparesis was present. Brain MRI showed globus pallidus central hyperintensity with surrounding hypointensity on T2-weighted images (eye of the tiger sign). Genetic analyses performed in the Institut für Humangenetik, Munich, Germany (Dr Monika Hartig, Prof. Thomas Meitinger), showed that the patient was a carrier of two typical *PANK2* mutations: c.1561G>A/p.Gly521Arg and c.1583C>T/p.Thr528Met. At the age of 18 the patient was successfully treated with bilateral GPi-DBS. Truncal dystonia subsided and the erect posture returned, he was able to walk with walker again. The severity of dystonic movements of the limbs was significantly reduced. The parents and two siblings of the patient 4 were tested for the *THAP1* mutation. His 7 years old brother remains asymptomatic. The proband’s 52-year-old clinically non-affected father was a carrier of the -249C>A substitution. This substitution was not found in the patient’s 20-year-old asymptomatic brother and in his 17-year-old symptomatic sister. She developed dystonia at the age of 7 and she was diagnosed as a carrier of the same *PANK2* mutations. Her dystonia started in feet and within three years spread to upper limbs. At the age of 17 she presented generalized dystonia involving all extremities, trunk, neck and face and features of spastic paraparesis.

The paternal grandfathers of the patient 4 never suffered from any involuntary movements. His father has five healthy siblings (2 sisters and 3 brothers), but they refused to consent to genetic testing.

Using the Mutation Taster *in silico* analysis, the coding Glu56Gly and Ile80Val substitutions were predicted as disease causing (probability 0.999 and 0.990, respectively). In contrast, the PolyPhen-2 HumVar model indicated these variations as benign with a score of 0.288 and 0.129, respectively. Both mutations were also predicted as tolerated with the use of SIFT algorithm.

Apart from the mutations described above, we analyzed the two-nucleotide -237-236 GA>TT substitution, consisting of two SNPs (rs370983900 and rs1844977763). Eleven out of 96 patients (11.5%) carried a heterozygous GA/TT genotype. The same genotype was identified in 7/150 (4.7%) controls. All remaining individuals were homozygous for the -237-236 GA genotype. The distribution of genotypes did not significantly differ between cases and controls (p = 0.076).

Moreover, one healthy control carried a heterozygous C/T genotype of rs146087734 silent single nucleotide variation in exon 1 (c.57C>T, p.Pro19Pro). This substitution was not found in the patients with dystonia.

## Discussion

In this study we report on *THAP1* mutations frequency of 4.2% (4/96 screened patients). The mutation frequency in our group was slightly higher but comparable with results obtained by other authors in unselected primary dystonia or DYT1-negative dystonia patients (1–2.5%) [8,16,17], however much lower than reported in groups of subjects restricted by age or family history (e.g. 25% in patients with onset under 22 and a positive familial history) [18].

Apart from the four probands, DYT6 mutations were found also in their relatives, including symptomatic as well as asymptomatic individuals. The mutation penetration was 66.7% (two affected individuals / three mutation carriers) in families of the patient 1 and patient 2, and 50% (two/four) in family of the patient 3. These results are close to the penetration range of 40–60% described in other studies [3].

In the family of the patient 4 the *THAP1* mutation penetration was 50% (one affected individual / two substitution carriers). However, the patient 4 was also a carrier of *PANK2* mutations and he developed phenotype characteristic for neurodegeneration with brain iron accumulation 1 (NBIA1). Moreover, the proband’s symptomatic sister carried the *PANK2*, but not *THAP1* mutation. Therefore it remains unknown if the -249C>A substitution has any influence on the dystonia phenotype.

The same Ile80Val substitution was found in patient 1 and patient 2. The mutation in the patient 2 was detected independently by LeDoux et al. [15] and in the present study. Both patients’ families lived in the neighboring regions of south Poland. We have no data confirming any distant relationship between these patients, however it could not be ruled out. The same mutation was also described in a German report [19].

The phenotypes of DYT6 patients reported before showed a high variability from focal to generalized dystonia. However the predominating phenotype was characterized by early onset and primary involvement of upper limbs or cranial-cervical region with progression to segmental or generalized dystonia. The presence of laryngeal or/and lower face dystonia was commonly described. Late onset was usually related to restriction of symptoms to one body region [8,18,20,21]. In all our *THAP1* mutation carriers (four probands and their three affected relatives) the first signs of dystonia occurred before the age of 23. The mean age of onset for seven subjects was 14.7 years. A primary localization typical for DYT6 dystonia was observed in six individuals. Five subjects (patient 1 and his sister, patient 2 and his son, the paternal uncle of patient 3) developed the first signs of dystonia in the upper limb. Three of them initially presented with the writer’s cramp. In patient 2 the disease began from laryngeal involvement. An uncommon primary involvement of the lower limb was noted in patient 4 (*THAP1* and *PANK2* mutations carrier).

Two of the identified substitutions, Glu56Gly and Ile80Val, are located within the THAP domain, essential for DNA-binding properties of the THAP1 protein. The Glu56 forms part of the H2 helix, which is involved in the zinc-binding domain formation. The Glu56Gly mutation has already been described in our previous paper, for more discussion see [10]. The Ile80 is located in the H4 helix in the C-terminal region of the THAP domain and belongs to the highly conserved AVPTIF motif, encompassing amino acids 76–81 [1]. *In silico* analyses of *THAP1* mutations, performed using different programs, often brought ambiguous results [22]. Here, both coding substitutions were predicted as harmful with the Mutation Taster, and as tolerated, with PolyPhen-2 and SIFT algorithms. According to the GeneBank SNP database the Ile80Val substitution has been described as a single nucleotide variation (SNV) and assigned the number rs372080941. Its allelic frequency is 4/121396 alleles (0.00003295), as reported in the Exome Aggregation Consortium database [23]. Recent reporter gene assay experiments supported a benign character of the Ile80Val mutation: this substitution was shown not to influence the THAP1 activity [19]. These results are consistent with a relatively benign character of the phenotype. This mutation was previously described by Lohmann et al. in a patient suffering from a focal cervical dystonia which had occurred when he was 41 years old. His family history was negative. In the case reported by Lohman et al. dystonia was limited to the neck [19]. In our subjects with this mutation the course of disease was more severe. Both Ile80Val carriers (patients 1 and 2) developed multifocal dystonia with almost identical distribution of the symptoms (Table 2) but with different age of onset (patient 1, 7 years; patient 2, 23 years). It should be noted that the Ile80Val variation was found only in dystonia patients and their relatives, but not in unrelated healthy controls, as reported in [15], [19] and the present study. Therefore, its clinical relevance could still be considered.

The third mutation found in the Polish dystonia cohort is the novel -249C>A substitution (patient 4). This variation is located within the 480-bps minimal *THAP1* promoter sequence that has recently been identified by Erogullari et al. [24]. Its localization may suggest a possible influence on the *THAP1* gene expression, however, functional analyses are necessary to confirm this assumption. The patient 4 carried not only *THAP1*, but also *PANK2* gene mutations. To our knowledge this is the first identified case carrying mutations in both genes. The patient’s -249C>A substitution-negative sister who is *PANK2* gene mutation carrier presented NBIA1 phenotype similar to the patient 4, whereas his -249C>A substitution positive father has not developed dystonia until the age of 52.

The patient presented with a rather typical pantothenate kinase 2-associated neurodegeneration (NBIA1/*PANK2*) phenotype [25]. In this subject dystonia developed first in the lower limbs. Initial involvement of the lower limb in DYT6 dystonia has already been reported, but it was not common [4–8]. The clinical picture of DYT6 dystonia and NBIA1 neurodegeneration has common features: childhood onset, involvement of limbs, trunk and larynx, the occurrence of dystonic tremor and writer’s cramp, slow progression of symptoms and a tendency to generalization. Therefore analysis of the patient’s 4 phenotype cannot be of help in establishing a possible clinical significance of the -249C>A substitution.

The silent variation in exon 1: c.57C>T, p.Pro19Pro (rs146087734) was previously described in one case of segmental craniocervical dystonia [21]. Here we identified this substitution only in one healthy control, but not in dystonia patients. Therefore, we cannot confirm its relevance as a causative agent for the disease. It should be classified rather as a rare benign variation.

The 5’UTR dinucleotide GA>TT variant was first described to increase risk for dystonia, however, this association was not confirmed in subsequent studies [19]. In our population, the GA/TT heterozygotes seemed to be slightly more frequent among the patients compared to the controls, however, this difference was not statistically significant.

It is of note that a large number of dystonia patients, especially cervical cases, harbor genetic variants defined as benign with the use of *in silico* methods. Therefore, it is not clear whether these variants are pathogenic or have no connection to the disease [2]. One possible explanation is that the benign variations may be in linkage disequilibrium with some harmful, not yet identified genetic alterations. The genetic background of DYT6 dystonia is additionally obscured by a low mutation frequency: at least 75% of patients screened in different studies, showing symptoms corresponding to DYT6 phenotype, harbor no *THAP1* mutations. On the other hand, genetic variants found in the patients are often present also in their asymptomatic relatives, and approximately 40% of dystonia cases have no familial history of the disease [26]. The latter phenomenon can be explained by a low penetrance. Another possibility is that the *THAP1* mutations, albeit associated with dystonia, are not sufficient to cause the disease. It was proposed that additional genetic or environmental factors are required for symptoms to manifest [2,27]. This concept brings us close to a multifactorial rather than a pure mendelian autosomal dominant mode of inheritance. This way, *THAP1* would be a part of a much bigger picture which other elements still remain to be discovered.
